# Family Functioning Affected by Adolescent Idiopathic Scoliosis in China: A Cross-Sectional Study

**DOI:** 10.3389/fped.2022.880360

**Published:** 2022-07-06

**Authors:** Yue Huang, Fuze Liu, Dejin Gao, Hai Wang

**Affiliations:** ^1^Department of Orthopaedic Surgery, Peking Union Medical College Hospital, Peking Union Medical College and Chinese Academy of Medical Sciences, Beijing, China; ^2^Department of Plastic and Reconstructive Surgery, Beijing Plastic Surgery Hospital, Peking Union Medical College and Chinese Academy of Medical Sciences, Beijing, China

**Keywords:** family functioning, mental health, McMaster family assessment device (FAD), adolescent idiopathic scoliosis (AIS), spinal deformity

## Abstract

Adolescent idiopathic scoliosis (AIS) is a common chronic disease in youths, presenting with spinal deformity. Previous studies reported that the family functioning of family members would be affected after a child is diagnosed with a chronic health condition. However, no previous study focused on the relationship between AIS and family function. This study is a cross-sectional study that enrolled 54 AIS families and 92 ordinary families and evaluated their family function in 7 domains using the McMaster family assessment device (FAD). The results showed that the AIS family got a lower score than a healthy family in all 7 subscales except for the problem-solving subscale. There was no significant difference between the patients with AIS (1.90 ± 0.42∼2.23 ± 0.32) and their parents (1.92 ± 0.35∼2.21 ± 0.29) in all seven subscales (*p* ≥ 0.05). The scores of the parents were moderately/strongly correlated with those of the patients with AIS in all seven subscales (γ = 0.456∼0.696, *p* < 0.05). Approximately, 20.4–87% of the families experienced unhealthy family functioning, with affective involvement (57.4%), and behavior control (87%) representing the unhealthiest subscales with the mean scores above the cutoff. It can be concluded that the AIS family performed better than a healthy family in family functions.

## Introduction

Adolescent idiopathic scoliosis (AIS) is the most common spinal deformity with a prevalence rate of 2–3% (1, 2). AIS is defined as a lateral curvature of the spine of unknown causes with a Cobb angle of more than 10 that occurs in adolescents aged 10–18 years (3). It usually aggravates during adolescence before skeletal maturation. AIS, a chronic illness, not only causes the patient cosmetic deformity but also alters their mental health and lifestyle (4–6). In addition, AIS can also affect the family, such as causing parental anxiety and depression in the patient, increasing the burden on the family budget, and altering intrafamilial relationships.

Family functioning indicates the ability of families to cope with stress (7). It is also one of the indicators of the family’s mental health and quality of life. When a child is diagnosed with a chronic health condition, the family functioning of its members will be affected (8). Meanwhile, the family also affects the child’s chronic health condition in turn. Therefore, it is quite necessary to measure family functioning to better understand the family’s responses to the child’s chronic health condition. To measure it, several tools have been developed (9). Among them, the McMaster family assessment device (FAD), first proposed in 1983, is one most widely used self-report questionnaires for both clinical and non-clinical individuals and families (10, 11). It has been translated into 27 languages for different populations and different cultures, including Chinese (11, 12).

Adolescent idiopathic scoliosis, as a chronic health condition, may affect the family functioning of the patient and their parents. Impaired family functioning may have a potential impact on the adolescent patient’s medical condition and treatment choice. Therefore, it is necessary to ➀ figure out whether AIS families suffer from a worse family function than ordinary families according to FAD, ➁ access the FAD subscales for both patients with AIS and their parents, ➂ investigate the relationship of the FAD subscales between patients with AIS and their parents; and ➃ identify the potential risk factors of the impaired FAD subscales.

## Materials and Methods

### Samples

This cross-sectional survey was conducted in the Department of Orthopaedic Surgery and Department of Rehabilitation in the authors’ hospital between April 2018 and April 2019. A total of 54 families with a child (aged from 12 to 17 years) diagnosed with AIS were finally enrolled in this survey. Each family had one patient and one matched parent participating in the study. We enrolled 92 healthy kids not suffering from chronic disease (aged from 12 to 15 years) from the high school affiliated to China Agricultural University and their one-matched parent in the study as the control group. The characteristics of the samples are shown in the flowchart in Supplementary Table 1. All participants were orally informed, and the parents signed informed consent. This study was approved by the ethics committee in the author’s hospital.

### Procedures

The questionnaires were completed in the AIS group when waiting for surgery or before undergoing exercise treatment. General demographic and familial structure questions were answered by parents after they completed the FAD. All image data were measured by two spinal surgeons based on x-ray images. In the control group, the questionnaires were completed in the Psychology Education course.

### Instruments

To assess family functioning, we used the modified FAD in Chinese, which is a 60-item self-report measure scored on a four-point scale (1∼4 points: from strongly agree to strongly disagree) (12). It consists of 7 subscales, including problem-solving (the family’s ability to resolve problems), communication (the exchange of information among family members), roles (patterns of behavior for handling basic family functions and whether these functions are clearly and equitably assigned), affective responsiveness (the ability of family members to experience appropriate affect), affective involvement (the extent of the interest of family members in each other), behavior control (the way in which a family expresses and maintains standards for the behavior of its members), and general functioning (the overall health of the family) (10). The questionnaire is designed to apply to family members over the age of 12 (13). These subscales have excellent internal consistencies (0.72∼0.92) and test–retest reliabilities (0.66∼0.76) (10, 14).

### Statistical

All data were described as means ± standard deviations or proportions. The relationship of 7 FAD subscale scores between the AIS group and the control group was evaluated by an independent sample *t*-test. The relationships of 7 FAD subscale scores between the patients with AIS and their parents were first accessed using a Student’s *t*-test since a previous study showed that, with the different life stages of individuals, the agreement on some scales of family function was found to be low (11). Family FAD subscale scores were defined as the mean scores of the patients and their parents. They were used to discriminate between “healthy” and “unhealthy” family functioning according to the cutoff values established by Miller et al. (14). In clinical practice, the Family FAD subscale was considered unhealthy if the Family FAD subscale scores were above the cutoff (problem-solving, communication, roles, affective responsiveness, affective involvement, behavior control, and general functioning’s cutoff is 2.2, 2.2, 2.3, 2.2, 2.1, 1.9, and 2, respectively). The potential risk factors of the unhealthiest subscales were first analyzed using the chi-squared test for the categorical data and the independent *t*-test for the measurement data. Then, the effects of independent risk factors were analyzed using the logistic regression model if more than two risk factors were identified. All the data were analyzed using IBM SPSS (version 22.0), and a *p-value* < 0.05 was considered statistically significant.

## Results

### General Characteristics

In the AIS group, a total of 54 families, including 54 patients with AIS (female:male = 51:3) and 54 parents (female:male = 40:14), were enrolled in this study. The mean ages of the patients and their parents were 14.9 ± 1.9 and 43 ± 4.1 years old, respectively. The mean Cobb angle of the major curve is 43.3° ± 17.8°. In control group, 92 healthy children (age = 13.5 ± 1.1 years old; F:M = 42:50) and their parents (age = 44.2 ± 4 years old; F:M = 52:40) were enrolled in this study. The other general characteristics of the samples are summarized in Supplementary Table 1. Though adolescent age is statistically significant, it can be ignored in clinical practice.

### Family Assessment Device Results

First, we tested whether AIS families suffer from a worse family function than ordinary families; the mean FAD subscale domain score of children and parents in the AIS group and the control group is shown in Supplementary Table 2. In general, AIS families got a lower score than families not suffering from chronic disease in communication, roles, affective responsiveness, affective involvement, behavior control, and general functioning domains. It seems that AIS families have better family functions in these domains.

Then, we focused on the AIS family. The mean FAD scores of both patients with AIS and their parents are shown in Table 1. There was no significant difference between the patients with AIS (1.90 ± 0.42∼2.23 ± 0.32) and their parents (1.92 ± 0.35∼2.21 ± 0.29) in all seven subscales (*p* ≥ 0.05). All seven subscale scores of the parents were moderately/strongly correlated with those of the patients with AIS (γ = 0.46∼0.70, *p* < 0.05). Family FAD scores, the average scores of the patients and their parents ranged from 1.91 ± 0.36 to 2.22 ± 0.29. Approximately, 20.4–87% of the families experienced unhealthy family functioning in subscales, such as problem-solving (20.4%), communication (37.4%), roles (31.5%), affective responsiveness (42.6%), affective involvement (57.4%), behavior control (87%), and general functioning (38.9%). Affective involvement (2.13 ± 0.31 > 2.1) and behavior control (2.22 ± 0.29 > 1.9) were the unhealthiest subscales with the mean scores above the corresponding cutoffs for disruption.

**TABLE 1:** The summarization of 7 FAD domains in AIS family.

Domain	Mean (SD) Patients parents Parents mean (SD)		*p*-value	Correlation coefficient (*P-value*)	Average (SD)	Cut-off	Above cut-off
Problem solving	1.98 (0.42)	1.98 (0.38)	0.50	0.64 (<0.00)*	1.98 (0.36)	2.2	20.4%
Communication	2.12 (0.42)	2.05 (0.38)	0.18	0.47 (<0.00)*	2.09 (0.34)	2.2	37.4%
Roles	2.19 (0.31)	2.21 (0.29)	0.36	0.63 (<0.00)*	2.20 (0.27)	2.3	31.5%
Affective responsiveness	2.15 (0.46)	2.19 (0.49)	0.33	0.60 (<0.00)*	2.17 (0.43)	2.2	42.6%
Affective involvement	2.11 (0.34)	2.15 (0.39)	0.29	0.46 (<0.00)*	2.13 (0.31)	2.1	57.4%
Behavior control	2.23 (0.32)	2.20 (0.34)	0.31	0.52 (<0.00)*	2.22 (0.29)	1.9	87.0%
General functioning	1.90 (0.42)	1.92 (0.35)	0.39	0.70 (<0.00)*	1.91 (0.36)	2.0	38.9%

*P < 0.05 considered as statistical significance.

### Risk Factors

We use the chi-squared test or the independent *t*-test when appropriately to find out risk factors that make family suffer from worse family function in affective involvement and behavior control subscale (Table 2). In the affective involvement subscale, coronal imbalance and brace treatment were significantly associated with unhealthy families (*p* = 0.014 and 0.037, respectively). The binary logistic regression results further revealed that only the education of the patient’s mother has contributed to better performance on the affective involvement subscale [*p* = 0.04, Odds ratio (OR) = 0.14] (Supplementary Table 3). No risk factor was identified for unhealthy families in the behavior control subscale.

**TABLE 2:** Risk factors of AIS families in affective involvement and behavior control subscales disrupting.

	Affective involvement			Behavior control		
	Disrupted (*n* = 31)	Healthy (*n* = 23)	*P*	Disrupted (*n* = 47)	Healthy (*n* = 7)	*P*
Ages of patients (years old)	15.29 (1.83)	14.35 (1.82)	0.03*	15.00 (1.91)	14.14 (1.46)	0.13
Course of disease (months)	21.71 (22.51)	15.09 (20.90)	0.14	19.44 (22.12)	15.14 (21.47)	0.32
Frontal balance (mm)	13.03 (11.76)	8.30 (7.71)	0.04*	11.40 (11.05)	8.43 (3.55)	0.24
Cobb angle of major curve (°)	46.32 (14.63)	39.22 (20.97)	0.07	43.74 (18.07)	40.29 (16.72)	0.32
Brace application Yes	12	3	0.04*	13	2	0.63
No	19	20		34	5	
Shoulder height Equal	14	18	0.01*	26	6	0.13
Unequal	17	5		21	1	
Health insurance Yes	24	19	0.45	36	7	0.18
No	7	4		11	0	
Residence city Yes	26	21	0.35	41	6	0.65
No	5	2		6	1	
Live with parents YES	30	22	0.68	45	7	0.75
No	1	1		2	0	
Marriage of parents married	28	21	0.90	42	7	0.49
Not married	3	2		5	0	
House hold income						
≤ ¥4,000/month	10	4	0.22	13	1	0.43
¥4,000–8,000/month	14	9		20	3	
≥ ¥8,000/month	7	10		14	3	
Father’s education college	14	11	0.85	21	4	0.41
Others	17	12		26	3	
Mother’s education college	7	12	0.02*	16	3	0.47
Others	24	11		31	4	

*P < 0.05 considered as statistical significance (in bold values). Healthy and disrupted was defined by the cut-off.

## Discussion

Analyses of FAD scores in this current study showed that, in general, families who suffered from AIS seem to have better performance on family functions in most domains. This may be due to AIS families having the same goal in their daily life to overcome difficulties, which makes the whole family get united. On the other hand, families that have not suffered from the chronic disease may ignore other members’ feelings in their daily life, resulting in poor performance in family functions.

When focusing on the AIS family, there was no significant difference between the patients with AIS and their parents. Moderate or strong agreements on all seven subscales were found between patients with AIS and their parents (Table 1). It demonstrated that parents and their adolescent patients had similar experiences of family functioning. For both of them, the FAD subscales with dichotomized answer categories can provide psychometric valid estimates of family functioning (9). Therefore, a family FAD score can be defined as the average score of the adolescent patient and their parent.

The interaction between an individual member and the family system, crucial to the maintenance of family health, is complex and operates in both directions (15). Once a child is diagnosed with a chronic health condition, the family will be affected (8). Meanwhile, the family will also affect the child’s health condition and *vice versa* (8). Family functioning plays a central role in keeping family members’ health, disease prevention, and health promotion of families. Unhealthy family functioning raises the risk of significant emotional and behavioral problems in children and is associated with worse treatment outcomes. This study indicates that some family functioning dimensions of 20.4∼87% of families were disturbed in AIS families. Therefore, understanding the impact of AIS on families is essential for family empowerment and the development of medical interventions and family support.

Among 7 FAD subscales, affective involvement and behavior control were the unhealthiest subscales with the mean family score above the suggested healthy cutoff and more than 50% of unhealthy families. Affective involvement is concerned with the extent to which family members are interested in and place value on each other’s activities and concerns (10). It refers not only to what the family does together but also to the level of participation between family members (13). The healthiest families have intermediate levels of involvement, and either too little or too much will contribute to a high (unhealthy) score of affective involvement (15). Therefore, this result disclosed that over half of the AIS families experience difficulties expressing and being receptive to others’ emotions between adolescent patients and their parents after a child is diagnosed with AIS.

To find the risk factors of unhealthy families in affective involvement, ages of the patients, coronal imbalance, brace treatment, frontal balance, and mother’s education experience were identified using univariate analysis. However, the result of the multivariate further confirmed that all these factors, except for the mother’s education experience, were confounding factors, and only the mother’s education experience proved to be an independent risk factor. This finding agrees with previous studies, which showed that brace treatment is not related to serious psychosocial difficulties (16–18). Also, a mother’s education experience seems to be crucial to children’s mental health. The coronal imbalance may reduce patients with AIS’ satisfaction with the body image, leading to low self-esteem, psychological distress, and impairment in daily functioning (17, 19), but in our study, the coronal imbalance is not an independent factor that affects family function in affective involvement. Among patients whose mothers have better educational experience, the patients may be inspired to express their own feelings and accept others’ emotions, which causes a healthier score of affective involvement function.

Behavior control mainly assesses the way in which a family expresses and maintains standards for the behavior of its members (10). It reflects the extent to which the family creates and abides by a certain set of rules governing appropriate member behavior (20). Poor behavior control can be associated with emotional issues (anxiety and depression) in children and their parents (20, 21). Previous studies suggested that the emotion of patients with AIS and their parents can be negatively affected by AIS (5, 22). It may explain why behavior control is the worst FAD subscale for AIS families (the most proportion of unhealthy families). The relationship between emotional issues and poor behavior control should be evaluated in the future. This study has not figured out the possible factors that ruin the family’s behavior control, but it may be due to the sample size being limited in this study.

Concerning the limitations of this study, only one parent was included in this study, and it could not illustrate the relationship of family functioning between fathers and mothers. Also, the emotional issues of family members were not evaluated in this study. Thus, it could not further offer more relationships between emotion and family functioning. Finally, the sample size was still limited, and a multi-center study may be further performed in the future.

## Conclusion

The AIS families have better performance in family functions than ordinary families. Family functioning presented a moderate/strong agreement between AIS children and their parents in families with patients with AIS. Approximately, 20.4∼87% of families reported unhealthy family functioning in seven FAD dimensions, especially for affective involvement and behavior control with over half of the unhealthy families. Mother’s college education was an independent protecting factor of affective involvement. These facts suggest a potential need of the family for assistance, especially for the patient’s mother who has poor education experience. Therefore, medical workers should provide some practical suggestions for AIS families to improve their family functioning when they come to see a doctor, such as praising the child’s good behavior, maintaining respect for each other, and strengthening communication. Moreover, this study figured out that families suffering from a poor family functioning are quite widespread in society since ordinary families seem to perform worse than the AIS families.

## Author Contributions

YH contributed to the acquisition of data, the analysis of data, statistical analysis, interpretation of results, and manuscript preparation. FL contributed to statistical analysis. DG contributed to the acquisition of data and data analysis. HW contributed to study design, study coordination, and manuscript preparation. All authors read and approved the final manuscript and made substantive intellectual contributions to this study to qualify as authors.

## Conflict of Interest

The authors declare that the research was conducted in the absence of any commercial or financial relationships that could be construed as a potential conflict of interest.

## Publisher’s Note

All claims expressed in this article are solely those of the authors and do not necessarily represent those of their affiliated organizations, or those of the publisher, the editors and the reviewers. Any product that may be evaluated in this article, or claim that may be made by its manufacturer, is not guaranteed or endorsed by the publisher.
