# Supplementary material for: Family Functioning Affected by Adolescent Idiopathic Scoliosis in China: A Cross-Sectional Study
Source: Front Pediatr. 2022 Jul 6;10:880360. doi: 10.3389/fped.2022.880360 (PMC9299375; doi:10.3389/fped.2022.880360)
Supplement: Supplementary file 1 [file Data_Sheet_1.docx]

Supplementary Table 1 The general characteristics in control group and AIS group

| Variables | Control group  (n=92) | AIS group  (n=54) | p |
| --- | --- | --- | --- |
| Age of children(years old) | 13.5(±1.1) | 14.9(±1.9) | 0.00* |
| Sex of children Male | 50(54.3%) | 3(5.6%) |  |
| Female | 42(45.7%) | 51(94.4%) |  |
| Brace application Yes | - | 15(27.8%) |  |
| No | - | 39(72.2%) |  |
| Cobb angle of the major curve(°) | - | 43.3±17.8 |  |
| Shoulder height Equal | - | 22(40.7%) |  |
| Unequal | - | 32(59.3%) |  |
| Age of parents (years old) | 44.2(±4.0) | 43.0(±4.1) | 0.08 |
| Sex of parents Male | 52(56.5%) | 14(25.9%) |  |
| Female | 40(43.5%) | 40(74.1%) |  |
| Health insurance (Yes) | 72(78.3%) | 43(79.6%) | 0.15 |
| Residence City | 82(89.1%) | 47(87.0%) | 0.30 |
| Marriage of parents Married | 83(90.2%) | 49(90.7%) | 0.08 |
| Divorced | 9(9.8%) | 5(9.3%) |  |
| Live with parents Yes | 85(92.4%) | 52(96.3%) | 0.65 |
| Household income  ≤¥4000/month | 26(28.3%) | 14(25.9%) | 0.14 |
| ¥4000~¥8000/month | 35(38.0%) | 23(42.6%) |  |
| ≥¥8000/month | 31(33.7%) | 17(31.5%) |  |
| Education of father  Primary school | 1(2.2%) | 1(1.8%) | 0.37 |
| Junior high school | 20(23.9%) | 11(20.4%) |  |
| Senior high school | 28(31.5%) | 17(31.5%) |  |
| University | 43(42.4%) | 25(46.3%) |  |
| Education of mother  Primary school | 1(1.1%) | 0(0%) | 0.11 |
| Junior high school | 33(21.7%) | 19(35.2%) |  |
| Senior high school | 26(30.4%) | 16(29.6%) |  |
| University | 32(46.7%) | 19(35.2%) |  |

* P<0.05 considered as statistical significance

Supplementary Table 2 FAD subscale score of AIS group and control group

|  | Control group | AIS group | t-test（p value） |
| --- | --- | --- | --- |
| problem solving | 2.00(0.50) | 1.98(0.42) | 0.31(0.38) |
| communication | 2.42(0.27) | 2.12(0.42) | 5.24 (<0.00)* |
| roles | 2.81(0.35) | 2.18(0.31) | 10.89(<0.00)* |
| affective responsiveness | 2.81(0.39) | 2.15(0.46) | 9.25(<0.00)* |
| affective involvement | 2.91(0.52) | 2.11(0.34) | 9.98(<0.00)* |
| behavior control | 2.69(0.28) | 2.23(0.32) | 9.13(<0.00)* |
| general functioning | 2.51(0.26) | 1.90(0.42) | 10.76(<0.00)* |

* P<0.05 considered as statistical significance

Supplementary Table 3. Binary logistic regression results of affective involvement disruption (Method = LR)

| Variables | p | Odd ratio (OR) | 95% confidence interval (CI) |
| --- | --- | --- | --- |
| Ages of patients | 0.62 | 1.63 | 0.76~1.58 |
| Frontal balance | 0.47 | 0.49 | 0.86~1.07 |
| Shoulder equal | 0.14 | 4.44 | 0.58~57.40 |
| Brace application | 0.08 | 5.81 | 0.85~20.29 |
| Mother’s college education | **0.04*** | 0.14 | 0.05~0.78 |

* P<0.05 considered as statistical significance
